# Oxytocin-Modulated Ion Channel Ensemble Controls Depolarization, Integration and Burst Firing in CA2 Pyramidal Neurons

**DOI:** 10.1523/JNEUROSCI.0921-22.2022

**Published:** 2022-10-12

**Authors:** Jing-Jing Liu, Katherine W. Eyring, Gabriele M. König, Evi Kostenis, Richard W. Tsien

**Affiliations:** ^1^New York University Neuroscience Institute, New York University School of Medicine, New York, New York 10016; ^2^Neurogenetics Program, Department of Neurology, David Geffen School of Medicine, University of California, Los Angeles, Los Angeles, California 90095; ^3^Institute for Pharmaceutical Biology, University of Bonn, Bonn 53113, Germany

**Keywords:** CA2, hippocampus, ion channel, neuromodulator, oxytocin, sodium channel

## Abstract

Oxytocin (OXT) and OXT receptor (OXTR)-mediated signaling control excitability, firing patterns, and plasticity of hippocampal CA2 pyramidal neurons, which are pivotal in generation of brain oscillations and social memory. Nonetheless, the ionic mechanisms underlying OXTR-induced effects in CA2 neurons are not fully understood. Using slice physiology in a reporter mouse line and interleaved current-clamp and voltage-clamp experiments, we systematically identified the ion channels modulated by OXT signaling in CA2 pyramidal cells (PYRs) in mice of both sexes and explored how changes in channel conductance support altered electrical activity. Activation of OXTRs inhibits an outward potassium current mediated by inward rectifier potassium channels (*I*_Kir_) and thus favoring membrane depolarization. Concomitantly, OXT signaling also diminishes inward current mediated by hyperpolarization-activated cyclic-nucleotide-gated (HCN) channels (*I*_h_), providing a hyperpolarizing drive. The combined reduction in both *I*_Kir_ and *I*_h_ synergistically elevate the membrane resistance and favor dendritic integration while the membrane potential is restrained from quickly depolarizing from rest. As a result, the responsiveness of CA2 PYRs to synaptic inputs is highly sharpened during OXTR activation. Unexpectedly, OXTR signaling also strongly enhances a tetrodotoxin-resistant (TTX-R), voltage-gated sodium current that helps drive the membrane potential to spike threshold and thus promote rhythmic firing. This novel array of OXTR-stimulated ionic mechanisms operates in close coordination and underpins OXT-induced burst firing, a key step in CA2 PYRs' contribution to hippocampal information processing and broader influence on brain circuitry. Our study deepens our understanding of underpinnings of OXT-promoted social memory and general neuropeptidergic control of cognitive states.

**SIGNIFICANCE STATEMENT** Oxytocin (OXT) plays key roles in reproduction, parenting and social and emotional behavior, and deficiency in OXT receptor (OXTR) signaling may contribute to neuropsychiatric disorders. We identified a novel array of OXTR-modulated ion channels that operate in close coordination to retune hippocampal CA2 pyramidal neurons, enhancing responsiveness to synaptic inputs and sculpting output. OXTR signaling inhibits both potassium conductance (*I*_Kir_) and mixed cation conductance (*I*_h_), engaging opposing influences on membrane potential, stabilizing it while synergistically elevating membrane resistance and electrotonic spread. OXT signaling also facilitates a tetrodotoxin-resistant (TTX-R) Na^+^ current, not previously described in hippocampus (HP), engaged on further depolarization. This TTX-R current lowers the spike threshold and supports rhythmic depolarization and burst firing, a potent driver of downstream circuitry.

## Introduction

Neuromodulators can alter neuronal intrinsic membrane properties and synaptic transmission, resulting in neuronal excitability changes to reshape circuit function and guide behavior (Marder, 2012; Vanoye et al., 2013). Oxytocin (OXT)/vasopressin nonapeptide family is highly conserved across evolution, with at least a dozen homologs in invertebrate and vertebrate taxa (Jurek and Neumann, 2018; Theofanopoulou et al., 2021). In mammals, OXT is primarily synthesized by hypothalamic neurons in the paraventricular (PVN) and supraoptic nuclei. In addition to the periphery, OXT neurons also project to central targets in the brain including the nucleus accumbens, septum, amygdala, and hippocampus (HP; Knobloch et al., 2012). CNS OXT has key roles in controlling reproduction, social behavior, and emotion (Ferguson et al., 2001; Baumgartner et al., 2008; Nishimori et al., 2008; Marlin et al., 2015), and has been proposed as a possible therapeutic for autism and schizophrenia (Penagarikano et al., 2015; Zik and Roberts, 2015; Eyring and Geschwind, 2021). Mammals express a single gene encoding the OXT receptor (OXTR), a G (guanine nucleotide-binding) protein-coupled receptor widely expressed in the brain (Mitre et al., 2016). Activation of OXTR generally depolarizes target cells and induces a variety of effects on synaptic transmission, with varied signaling cascades and ionic mechanisms suggested (Tomizawa et al., 2003; Wang and Hatton, 2007; Owen et al., 2013; Jiang et al., 2014; Tang et al., 2014; Briffaud et al., 2015; Tirko et al., 2018; Maniezzi et al., 2019; Hu et al., 2020, 2021; Zhang et al., 2021). In the HP, OXTRs express at high levels in the CA2 and CA3a subregions (Mitre et al., 2016; Tirko et al., 2018). Dorsal CA2 (dCA2) is demonstrated to be critical for HP-dependent brain oscillations (Oliva et al., 2020) and social memory formation (Hitti and Siegelbaum, 2014; Raam et al., 2017; Oliva et al., 2020; Lopez-Rojas et al., 2022). Our previous study found that OXTR mediated signaling depolarizes and induces burst firing in dCA2 PYRs (Owen et al., 2013; Tirko et al., 2018). Thus, clarifying the ionic mechanism of OXT modulation of dCA2 PYR firing is important for understanding how OXT controls HP circuitry and social memory.

In the present study, we aimed at a comprehensive survey of the ionic mechanisms whereby activation of OXTRs might modulate CA2 neuron intrinsic properties. Our results demonstrate that activation of OXTRs inhibits the inward rectifier K^+^ channels (*I*_Kir_), depolarizing the cell from its resting potential. At the same time, OXT signaling also suppresses the hyperpolarization-activated cyclic-nucleotide-gated (HCN) channel mediated current (*I*_h_), which drives cell hyperpolarization. These opposing forces restrain the membrane potential from being depolarized quickly, while synergistically elevating membrane resistance and thus favoring dendritic integration, reflected by enlarged mini EPSCs.

Unexpectedly, we further identified an inward tetrodotoxin-resistant (TTX-R) sodium current in CA2 PYRs that is activated by OXTR activation, whereas OXT spared the conductance contributed by Na^+^ leak channels, two-pore-domain K^+^ channels, Ca^2+^ or Cl^–^ channels. In combination, this novel array of OXTR-stimulated ionic mechanisms strongly elevates the responsiveness of CA2 PYRs toward synaptic inputs and promotes burst firing.

## Materials and Methods

### Slice preparation

Experimental protocols were approved by the Institutional Animal Care and Use Committee of New York University Grossman Medical School. Mice one to two months old with both sexes were anesthetized with a mixture of ketamine/xylazine (150 and 10 mg/kg, respectively) and perfused transcardially with an ice-cold sucrose solution containing (in mm): 206 sucrose, 11 D-glucose, 2.5 KCl, 1 NaH_2_PO_4_, 10 MgCl_2_, 2 CaCl_2_, and 26 NaHCO_3_, bubbled with 95% O_2_-5% CO_2_. Following animal perfusion and decapitation, brains were removed and placed in the cold sucrose for dissection. Because of the smaller size of the mouse brain, we prepared the transverse brain slices without dissecting out the HPs. After extracting the whole brain, the cerebellum and brain stem were removed, and the brain hemispheres were separated physically. A cut on each hemisphere was made on the side of the caudal HP with a scalpel blade. Then we positioned the two hemispheres vertically with the plane made by the cutting facing down onto a mounting block with glue, and transferred them to the sectioning stage of a Leica VT 1200S Vibratome. We typically prepared ∼350-µm sections from the rostral one-third of the HP containing the dCA2 region for physiology experiments. Cut sections of left and right HP were transferred to an oxygenated recovery chamber filled with artificial CSF (ACSF) containing (in mm): 122 NaCl, 3 KCl, 10 D-glucose, 1.25 NaH_2_PO_4_, 2 CaCl_2_, 2 MgCl_2_, and 26 NaHCO_3_, bubbled with 95% O_2_-5% CO_2_ at 34°C. After incubation, slices were held in bubbled ACSF at room temperature for up to 6 h until recordings were made.

### Electrophysiological recordings

For recording, slices were placed in a submerged slice chamber continuously perfused with ACSF at a rate of 1–3 ml/min and maintained at a bath temperature of 30°C. Tdtomato-positive neurons in the CA2 pyramidal cell (PYR) layer were visualized with LED illumination under an upright microscope. Whole-cell patch-clamp recordings were performed as described previously (Liu et al., 2017; Tirko et al., 2018), using a MultiClamp 700B amplifier (Molecular Devices) and pCLAMP version 10.7.0.2 for data collection. Signals were filtered at 10 kHz and sampled at 20–50 kHz with a Digidata 1440 data acquisition interface. Patch pipettes with a resistance of 3∼5 MΩ were made from borosilicate glass (World Precision Instruments) with a Sutter Instrument P-97 micropipette puller and filled with a solution containing (in mm): 126 K-gluconate, four KCl, 10 HEPES, 4 Mg-ATP, 0.3 Na_2_-GTP, and 10 phosphocreatine (pH to 7.2 with KOH) or a high Cl^–^ solution containing (in mm): 90 K-gluconate, 40 CsCl, 1.8 NaCl, 1.7 MgCl_2_, 3.5 KCl, 0.05 EGTA, 10 HEPES, 2 Mg-ATP, 0.4 Na_2_-GTP, 10 phosphocreatine, and 5 QX314 (pH to 7.2 with CsOH). Input resistance (Rin) and series resistance were monitored throughout the experiments, and recordings were rejected if series resistance increased to above 15 MΩ, or the initial resting potential was more depolarized than −62 mV. Pipette series resistance was compensated by 70% during voltage-clamp experiments. Fast and slow voltage ramps were used to determine current–voltage (*I-V*) curves approximating steady state, taking the mean current value after signal averaging over two to four trials. Liquid junction potential (12 mV) was not corrected.

### RNAScope *in situ* hybridization

To detect the mRNA of OXTR and tdTomato in OXTR::Ai9 mice, a standard protocol suggested by the manufacturer (Advanced Cell Diagnostics) was followed and the RNAScope Fluorescent Multiplex Reagent kit was used. Two male and two female mice were used for this experiment. RNAScope probes used were Mm-OXTR, tdTomato, Mm-Ppib (positive control probe) and DapB (negative control probe). In brief, fresh-frozen brain samples were obtained from wild-type animals, cut into 15-µm slices by cryostat and mounted on slides. Slides containing dorsal HP (dHP) were fixed (15 min, 4% paraformaldehyde) and dehydrated (50%, 70%, and 100% ethanol, 5 min each) before proceeding immediately to the RNAScope assay. OXTR and tdTomato were assigned to different fluorescent channels. Both positive and negative control probes were used to control the specificity of signals. Confocal images were taken using Zeiss LSM700 or LSM800. Two male and two female animals were used for each parameter. These experiments were not performed in a blinded manner.

### Drugs

All drugs were diluted in ACSF to the indicated final concentration and were bath applied. TGOT was obtained from Bachem. ZD7288 and XE991 were obtained from Cayman Chemical Company. VU 0134992, VU 590 dihydrochloride, Tertiapin LQ, Picrotoxin, ML133 hydrochloride, and CNQX were obtained from Alomone Lab. VU0314992 hydrochloride, repaglinide, and fluoxetine were obtained from Fisher Science.

### Experimental design and statistical analysis

The effect of OXTR activation was evaluated before and after the bath application of TGOT at concentrations ranging from 10 to 600 nm in the same neurons recorded in hippocampal brain slices, therefore these experiments were not performed blindly. In all cases, four or more animals with both sexes were used for each parameter collected and were pooled for analysis. Each recorded neuron came from one brain slice of one experimental animal. There was no repeated use of any brain slice. Individual sample sizes for slice patch clamp recording (*n* = number of neurons, included in each figure legend) are reported separately for each experiment. All statistical analysis was performed using GraphPad Prism 9. Statistical comparisons before and after the application of TGOT were made using paired two-tailed Student's *t* test. Statistical comparisons for different groups were made using one-way or two-way ANOVA and *post hoc* Tukey's test. Each statistical method is clearly stated in Results or the figure legends. All statistical tests were two-sided. Data distribution was assumed to be normal, but this was not formally tested. Data are presented as mean ± SEM. Individual data points are plotted in figures. All raw datasets are openly accessible on request.

## Results

### OXT increases the membrane excitability of CA2-OXTR^+^ neurons

**Figure 1.**
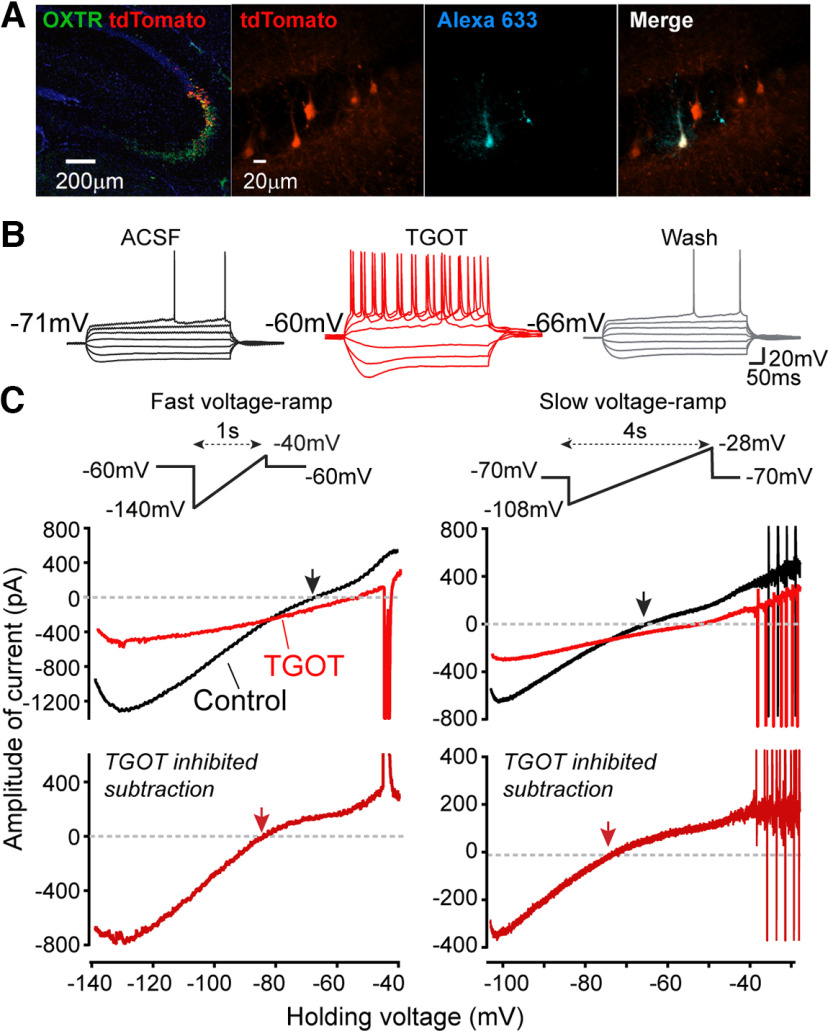
TGOT-induced current changes and neuronal excitability. ***A***, Left, mRNA expression of OXTR (green) and tdTomato (red). Right, Targeting of tdTomato^+^ neuron in HP CA2 region by whole-cell patch clamping in OXTR::Ai9 animals. Alexa Fluor 633 dye was included in the internal solution for some recordings when *post hoc* examination was needed. ***B***, Firing patterns of CA2 OXTR^+^ PYR induced by steps of current injection (−200Δ40pA) applied before, during application of TGOT (at the 25th min), and after washout (25-min wash). ***C***, Effect of TGOT on quasi-steady-state *I-V* relationship. Left inset, Fast voltage ramp (f-vr: −140 to −40 mV at 100 mV/s). Right inset, Slow voltage ramp (s-vr: −108 to −28 mV at 20 mV/s). Below, Corresponding whole cell currents, recorded both before (black traces) and during (red traces) application of 600 nm TGOT (at the 25th min). Upper, Currents plotted as a function of the command voltage. Lower, TGOT-inhibited current obtained by subtraction, shown as red traces. Downward spikes in current traces correspond to inadequately controlled APs.

OXTRs are highly expressed in many of the pyramidal neurons (PYRs) in the CA2 and distal CA3 regions of the dHP in the mouse brain (Mitre et al., 2016). To avoid possible intermingling of heterogeneous cell types of dCA2, we tried to specifically target OXTR-expressing (OXTR^+^) neurons in the middle of dCA2, using OXTR-cre mice crossed with an Ai9 tdTomato reporter line (Dudek et al., 2016; Raam et al., 2017; Young and Song, 2020). RNAscope *in situ* hybridization was performed in the offspring for validation using probes against mRNAs of OXTR and tdTomato, and both were found at high levels in the dHP of OXTR::Ai9 animals (Fig. 1*A*, left panel). In the pyramidal layer, the tdTomato (red) signals are highly restricted to area CA2, showing complete overlap with OXTR (green) signals (detectable OXTR mRNAs spread over wider areas in the HP compared with tdTomato; Fig. 1*A*, left panel). For the rest of this study, we performed whole-cell recordings from visually identified tdTomato^+^ cells in the central CA2 (Dudek et al., 2016), using both male and female adult animals. Characterization of intrinsic membrane properties was also performed at the beginning of each recording for further validation of typical CA2 PYR features by electrophysiological criteria (Chevaleyre and Siegelbaum, 2010; Hitti and Siegelbaum, 2014; Tirko et al., 2018; Robert et al., 2020, 2021). Alexa Fluor 633 dye was included in the internal solution for some recordings when *post hoc* recognition was needed (Fig. 1*A*, right panel).

In line with a previous report (Tirko et al., 2018), activation of OXTRs by [Thr^4^, Gly^7^]-OXT (TGOT), a highly specific OXTR agonist, strongly depolarized the CA2-OXTR^+^ neuron's resting membrane potential (*V*_m_, from −71.2 ± 1.3 to −59.0 ± 2.3 mV at 25th min of TGOT, *n* = 13, *p* < 0.0001), increased membrane resistance (R_m_, from 62.6 ± 2.3 to 79.8 ± 4.5 MΩ, *n* = 8, *p* = 0.0209). Spike amplitude decreased as already documented (Tirko et al., 2018). The peak levels of subthreshold changes driven by TGOT in *V*_m_ were generally reached during the 10th–25th min of agonist application inducing spontaneous action potential (sAP) firing in CA2-OXTR^+^ neurons, with a pattern dominated by spike clusters and bursts (Fig. 9*D*) (Tirko et al., 2018). In the presence of [1-D(CH^2^)_5_,Tyr(ME)^2^,Thr^4^,Tyr-NH_2_^(9)^] ornithine vasotocin (OTA) a selective OXTR antagonist, TGOT-induced changes in the *V*_m_ and R_m_ were blocked (Δ*V*_m_, 0.3 ± 1.3 mV, *n* = 4, *p* = 0.85; ΔR_m_, −11.8 ± 7.6 MΩ, *n* = 4, *p* = 0.21; comparing changes to a mean of zero using one sample *t* test), indicating a selective dependence on OXTR signaling. We also found that TGOT reduced the *V*_m_ threshold for evoking AP by current injections (from −42.6 ± 1.8 to −47.5 ± 1.0 mV, *n* = 10, *p* = 0.0017) and significantly enlarged the sag potential following a –200-pA hyperpolarizing current injection (from 1.8 ± 0.4 to 5.7 ± 1.4 mV, *n* = 7, *p* = 0.010; Fig. 1*B*). Because the sag is generated by HCN channel-mediated current (*I*_h_; Chevaleyre and Siegelbaum, 2010; Srinivas et al., 2017; Tirko et al., 2018; Robert et al., 2020), the enlargement could reflect an increase in the maximal *I*_h_ or a greater degree of hyperpolarization.

**Figure 2.**
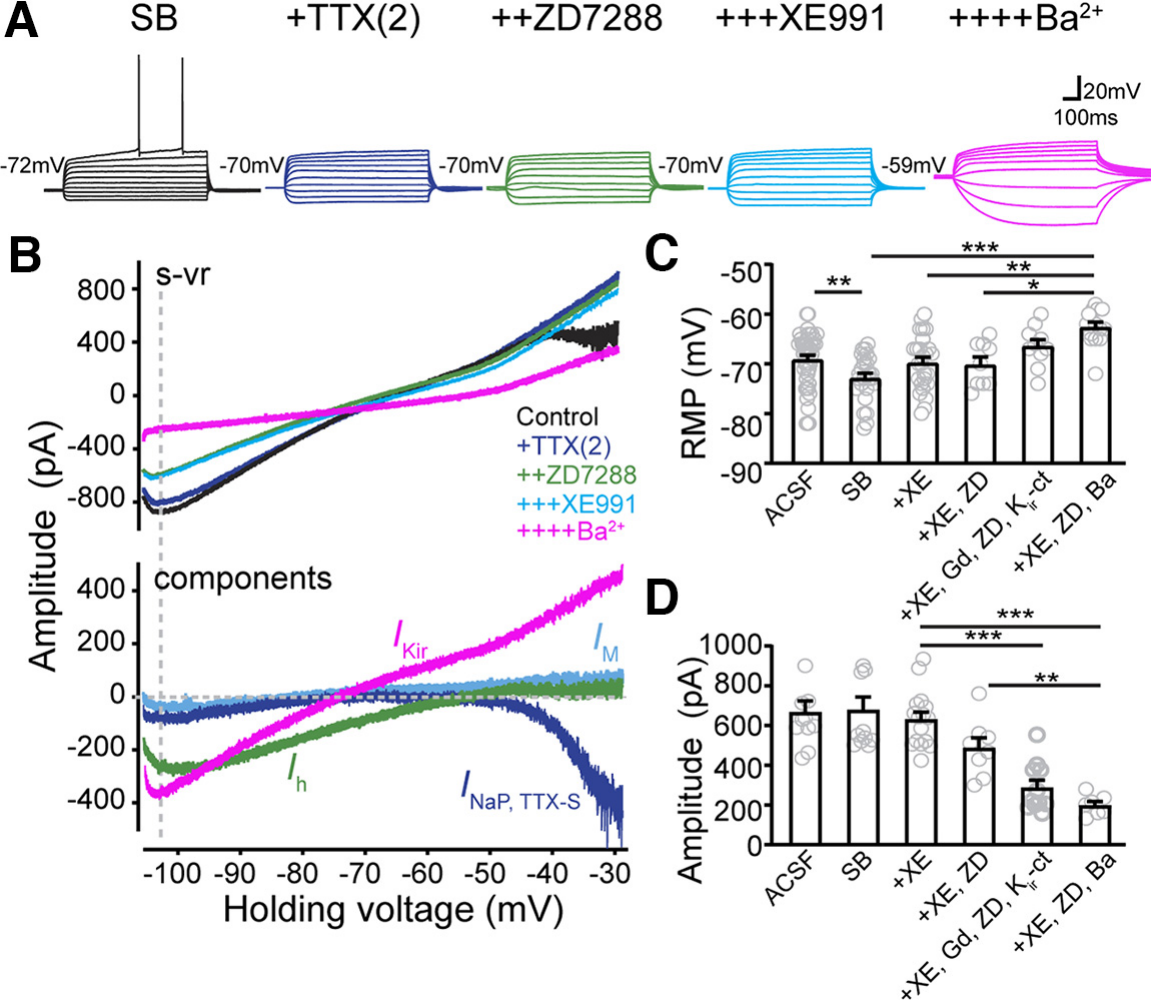
Identification of active current components in CA2 OXTR^+^ PYRs, including TTX-S persistent sodium current (*I*_NaP, TTX-S_), *I*_h_, M channel-mediated current (*I*_M_), and barium-sensitive K^+^ current (*I*_Kir_ and *I*_K2P_). ***A***, Responses of CA2-OXTR+ neurons in current-clamp step mode in a solution containing a cocktail of fast synaptic transmission blockers (SB: 10 µm NBQX, 25 µm D-AP5, 50 µm picrotoxin, and 1 µM CGP55845). Serial addition of 2 µm TTX [TTX(2)] to define *I*_NaP, TTX-S_ (purple), 20 µm ZD7288 (ZD) to define *I*_h_ (green), 10 µm XE991 (XE) to define *I*_M_ (blue), and 300 µm Ba^2+^ to define *I*_Kir_+*I*_K2P_ (pink). ***B***, Upper, *I-V* relationships evoked by s-vr recorded in the same cell under different conditions. Lower, Current components sensitive to each of the applied drugs were obtained by subtraction. ***C***, Pooled data of resting membrane potentials (RMPs) measured under current clamp with different drug additions. A cocktail of antagonists for K_ir_ channels (K_ir_-ct) was formulated (Table 1) and used to define *I*_Kir_. 1 mMGd^3+^ was used to define the leak sodium channel (*I*_NALCN_). Gray circles represent individual cells. Bar graphs represent mean ± SEM: ACSF, −67.4 ± 1.9 mV, *n* = 40; SB, −72.8 ± 0.9 mV, *n* = 26; SB+XE, 69.7 ± 1.1 mV, *n* = 26; SB+XE+ZD, −70.1 ± 1.5 mV, *n* = 9; SB+XE+ZD+Gd+K_ir_-ct, −66.4 ± 1.3 mV, *n* = 10; and SB+XE+ZD+Ba, −62.6 ± 1.0 mV, *n* = 14. *F*_(7,66)_ = 16.79, *p* < 0.0001, one-way ANOVA. *Post hoc* Tukey's tests for individual multiple comparisons: ACSF versus SB, *p* = 0.0356; SB versus SB+XE, *p* = 0.2292; SB+XE versus SB+XE+ZD+k_ir_-ct *p* = 0.0098; SB+XE versus SB+XE+ZD+Ba, *p* = 0.0005; SB+XE+ZD versus SB+XE+ZD+Ba, *p* = 0.0074. ***D***, Pooled data of amplitudes of currents measured at −105-mV command voltage (dashed vertical line in ***B***) during s-vr in solutions with different drug additions. Gray circles represent individual cells. Bar graphs represent mean ± SEM: ACSF, −667.6 ± 55.3 pA, *n* = 11; SB, −678 ± 65.1 pA, *n* = 11; SB+XE, −631.5 ± 35.2 pA, *n* = 16; SB+XE+ZD, 487.4 ± 51 pA, *n* = 8; SB+XE+ZD+Gd+K_ir_-ct, 288 ± 37.1 pA, *n* = 11; and SB+XE+ZD+Ba, 199 ± 19.1 pA, *n* = 7. *F*_(5,125)_ = 8.388, *p* < 0.0001, one-way ANOVA. *Post hoc* Tukey's tests for individual multiple comparisons: SB+XE versus SB+XE+ZD+k_ir_-ct, *p* < 0.0001; SB+XE versus SB+XE+ZD+Ba^2+^, *p* < 0.0001; SB+XE+ZD versus SB+XE+ZD+Ba^2+^, *p* = 0.009. *p < 0.05, **p < 0.01, ***p < 0.001.

In addition, the subthreshold membrane potential levels of CA2-OXTR^+^ neurons were found sensitive to spontaneous synaptic inputs. An inhibitory cocktail to block synaptic transmission via glutamate or GABA receptors (SB, containing 10 µm NBQX, 25 µm D-AP5, 50 µm picrotoxin, 1 µm CGP55845) negatively shifted *V*_m_ (from −69.15 ± 0.83 to −72.8 ± 0.9 mV, *n* = 39 and 26, respectively, *p* = 0.004; Fig. 2*C*). However, the presence of synaptic blockers did not affect the extent of TGOT-induced depolarization (from −74.3 ± 21.5 to −67.7 ± 1.9 mV, *n* = 7, *p* = 0.0011) or R_m_ increase (from 74.9 ± 2.3 to 84 ± 2.8 mΩ, *n* = 7, *p* = 0.0125. These results reaffirm that changes in intrinsic membrane properties, along with elevated synaptic drive, can contribute to OXTR-mediated enhancement of CA2 PYR excitability (Tirko et al., 2018).

**Table 1.** K_ir_ channel subtype mRNA expression level in dHP CA2 region

		Expression level			
Subtype	Gene	Mouse	Rat	Inhibitor	
K_ir_ 1.1	KCNJ1	*	*	Tertiapin LQ, VU 590 dihydrochloride	Estrada and Kaufman (2018); Lewis et al. (2009)
K_ir_ 2.1	KCNJ2	**	*	ML133 hydrochloride	Furst et al. (2014)
K_ir_ 2.2	KCNJ12	*	**	ML133 hydrochloride	Furst et al. (2014)
K_ir_ 2.3	KCNJ4	**	***	ML133 hydrochloride	Furst et al. (2014)
K_ir_ 2.4	KCNJ14	—	**	No inhibitor	
K_ir_ 3.1	KCNJ3	***	***	Tertiapin LQ	Estrada and Kaufman (2018)
K_ir_ 3.2	KCNJ6	***	***	Tertiapin LQ	Estrada and Kaufman (2018)
K_ir_ 3.3	KCNJ9	*	**	No inhibitor	
K_ir_ 3.4	KCNJ5	*	*	Tertiapin LQ	Estrada and Kaufman (2018)
K_ir_ 4.1	KCNJ10	—	*	VU 0134992	Zhang et al. (2021)
K_ir_ 4.2	KCNJ15	**	**	No inhibitor	
K_ir_ 5.1	KCNJ16	*	**	VU 0134992	Zhang et al. (2021)
K_ir_ 6.1	KCNJ8	—	*	Repaglinide	Wang et al. (2018)
K_ir_ 6.2	KCNJ11	**	**	Repaglinide	Wang et al. (2018)
K_ir_ 7.1	KCNJ13	N.A.		VU 590 dihydrochloride	Lewis et al. (2009)

*** high;

** medial;

* low; —, none; N.A., not applicable.

### Identification of overall conductance changed by OXTR signaling

To explore the ionic mechanisms underlying the neuronal excitability and firing behaviors induced by OXTR signaling, we first sought to identify the overall conductance change at subthreshold voltages in CA2 PYRs following TGOT stimulation. We performed interleaved voltage-clamp and current-clamp recordings in each neuron both before (control) and after TGOT application, which allowed a close correlation of the steady-state *I-V* relation with firing behavior. Spontaneous activity (I = 0) and activity during 1-s-long current pulses (−200Δ40pA) were recorded under current-clamp. The voltage-clamp recordings relied on imposed ramp waveforms as an efficient method for assessing the biophysical fingerprint of various current components (Yamada-Hanff and Bean, 2013). To provide coverage for ion channels with different voltage and time dependence, two standard ramp protocols were used: (1) fast ramp (f-vr, 100 mV/s) from −140 to −40 mV, and (2) slow ramp (s-vr, 20 mV/s) from −108 to −28 mV (for more details, see Materials and Methods). Figure 1*B*,*C* shows a typical experiment. The neuron had a stable resting potential of −71 mV under control conditions (black). Application of TGOT (red) depolarized *V*_m_ to −60 mV in 25 min, with increased spike number during current step injections, and the effects were largely reversed by 25 min wash (Fig. 1*B*, gray). Under control condition, the control *I-V* relationships showed net inward current over a negative voltage range with zero current intercepts (black arrows) near −70 mV (Fig. 1*C*, black traces), close to the resting *V*_m_ under current clamp. The f-vr evoked *I-V* curve showed a more obvious saturation of current near −130 mV, indicative of currents carried by the inwardly rectifying K^+^ (K_ir_) channels. At the 25th min exposure to TGOT (Fig. 1*C*, upper red traces), the inward current at strongly negative V_m_ was greatly reduced and the zero current intercepts were shifted rightward; the residual current was nearly linearly dependent on V_m_. The net TGOT-inhibited current was obtained by subtraction of the *I–V* curve in TGOT from that in control condition (Fig. 1*C*, lower panel). The TGOT-inhibited current displayed an inwardly rectifying *I–V* characteristic from −140 to roughly −60 mV, with a reversal potential (red arrows) of −80.3 ± 2.0 mV (*n* = 7), which would have approximated the K^+^ equilibrium potential (*E*_K_) had we chosen to correct for junction potential (∼12 mV; Tirko et al., 2018). Beyond −60 mV, the calculated difference current no longer showing inward rectification, but grew with a positive slope instead (Fig. 1*C*, lower panel). We interpreted this as reflecting a TGOT-induced inward current, possibly carried by depolarization-activated, sodium-permeable channels, as further documented below. Such current would cause an upward deflection in a plot of “TGOT-inhibited current.” This exemplar and many other recordings included AP-induced currents that escaped voltage-clamp control, presumably reflecting an inability to obtain space clamp of the axon initial segment. However, the trajectory of steady-state current was generally continuous before and after the escaped spikes, suggesting that the majority of the recorded steady-state current was under good voltage control (Goldberg et al., 2008; Yamada-Hanff and Bean, 2013). Our initial data (Fig. 1) suggested that TGOT modulation at subthreshold V_m_ might involve multiple components, including currents carried by K^+^ channels (e.g., K_ir_ channels) and cation channels.

### Endogenous ion channel conductance in CA2 PYRs

Because OXTR activation led to burst-like AP firing in CA2 PYRs, we next explored the repertoire of currents mediated by various ion channels in the CA2 neurons at basal level, focusing on current components previously associated with pacemaking. Based on precedent across a variety of neurons, these included TTX-sensitive (TTX-S) persistent Na^+^ current (*I*_NaP, TTX-S_), hyperpolarization-activated current (*I*_h_), depolarization-activated K^+^ current known as M-current (*I*_M_), and inward rectifier K^+^ current (*I*_Kir_; Yamada-Hanff and Bean, 2013). We performed interleaved voltage-clamp and current-clamp recordings in the same CA2-OXTR^+^ neurons during successive applications of 2 µm TTX [TTX(2)] to characterize *I*_NaP, TTX-S_ (Yamada-Hanff and Bean, 2013), 20 µm ZD7288 to capture *I*_h_, 10 µm XE991 to capture *I*_M_, and 300 µm Ba^2+^ to capture K^+^ currents including *I*_Kir_. During data collection, the brain slice was exposed to each antagonist for at least 15 min before applying the next one. An example of such an experiment is shown in Figure 2*A*,*B*. Under current clamp, the resting *V*_m_ of CA2 neurons showed a particular sensitivity to Ba^2+^, whereas other antagonists had no significant effect (Fig. 2*A*,*C*). Ba^2+^ also increased the R_m_ and induced repetitive oscillatory activity of unidentified origin during depolarization (Fig. 2*A*).

Each antagonist-sensitive current was obtained by subtraction of the after-treatment *I–V* curve from the before-treatment one (Fig. 2*B*, lower). In CA2 PYRs, *I*_NaP, TTX-S_ was first evident at −46.5 ± 1.0 mV (*n* = 7), a level more depolarized compared with *I*_NaP, TTX-S_ in HP CA1 PYRs (Yamada-Hanff and Bean, 2013). ZD7288-sensitive inward current conveyed by *I*_h_ was maximal near −105 mV and decreased with depolarization to near zero at −58.3 ± 1.3 mV (*n* = 6). In contrast, XE991-sensitive *I*_M_ was almost undetectable at voltages below −50 mV and remained small even with progressive depolarization. Applied last, Ba^2+^ inhibited a large conductance with an *I-V* relationship with a negative reversal potential and rectifying shape, roughly similar to the TGOT-inhibited current previously observed (Fig. 1*C*, lower). The Ba^2+^-sensitive inward current was the largest of the pharmacologically-defined current components in collected data taken at −105 mV (Fig. 2*B*,*D*; *F*_(5,125)_ = 8.388, *p* < 0.0001, one-way ANOVA). Thus, in the negative voltage range where TGOT showed a strong suppressive effect on inward current in CA2 PYRs, the dominant current components were carried by K_ir_ and HCN but not M channels.

### OXTR signaling inhibits current carried by k_ir_ channels

**Figure 3.**
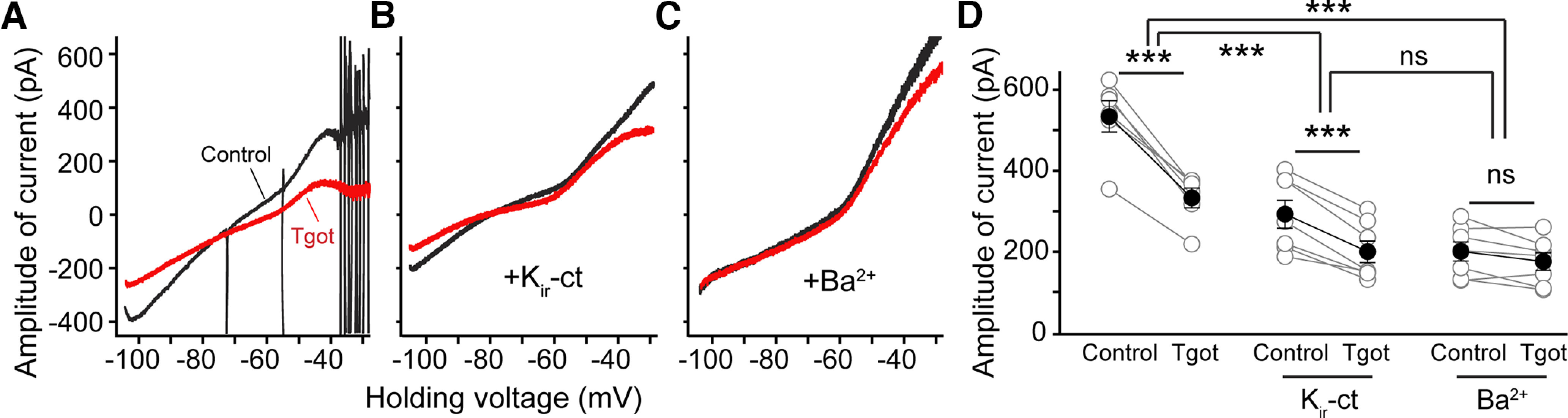
TGOT closes currents carried by K_ir_ channel. K_ir_-ct and Ba^2+^ partially or completely occlude TGOT-induced inhibition of inward current over negative voltage range. ***A***, Effect of TGOT on *I-V* relationship evoked by s-vr, recorded before (black) and during (red, at the 25th min) application of TGOT. Currents are plotted as a function of the command voltage. To eliminate influences of other ion channels possibly modulated by OXTR signaling, SB; XE991, ZD7288 were included in the external solution. ***B***, Effect of TGOT on *I-V* relationship measured as experiment A when SB, Gd^3+^, TTX(2), XE991, ZD7288, and K_ir_-ct were included in the external solution. ***C***, Effect of TGOT on *I-V* relationship measured as experiment A when SB, TTX(2), XE991, ZD7288, and 300 µm Ba^2+^ were included in the external solution. The *I-V* relationships measured in ***B***, ***C*** showed that both K_ir_ ct and Ba^2+^ largely reduced the inward current at baseline. Moreover, inhibition caused by further application of TGOT was strongly reduced or completely occluded by preexposure to K_ir_ ct or Ba^2+^, respectively, suggesting that inhibition of *I*_Kir_ likely accounts for the majority of the inward current suppression by TGOT. ***D***, Pooled data of amplitudes of currents measured at –105-mV command voltage under s-vr before and during TGOT. Gray dots represent individual cells. Black dots and error bars denote mean ± SEM *F*_(2,31)_ = 26.09, *p* < 0.0001, two-way ANOVA. Paired two-tailed Student's *t* tests were used for within group comparison: TGOT reduced current amplitude in control solution (SB, XE991, ZD7288) from 534 ± 38.5 to 333.2 ± 24.4, *n* = 6, *p* = 0.0003; in solution including K_ir_-ct from 292.7 ± 34.3 to 199.9 ± 26.8, *n* = 7, *p* = 0.0004; and in solution including Ba^2+^ from 200.6 ± 23.6 to 175.4 ± 21.7, *n* = 7, *p* = 0.07. ns: no significance, *p < 0.05, **p < 0.01, ***p < 0.001.

K_ir_ channel regulation has been implicated in other neuronal or non-neuronal cells subject to OXT neuromodulation (Jiang et al., 2014; York et al., 2017; Hu et al., 2020), but has not been considered as a target for modulation of CA2 PYR (Owen et al., 2013; Tirko et al., 2018; Robert et al., 2020). In our strategy to focus specifically on *I*_Kir_, we preblocked other subthreshold conductance (*I*_h_ and *I*_M_) and then compared the TGOT-sensitive current before and after inhibition of *I*_Kir_ (Fig. 3). Relative to the TGOT-induced current change with K_ir_ channels intact (Fig. 3*A*,*D*), the TGOT-sensitive current was significantly reduced, but not eliminated, by exposure to a cocktail of antagonists for K_ir_ channels (K_ir_-ct; Fig. 3*B*,*D*). K_ir_-ct was formulated to block as many of the known components of K_ir_ as possible (Table 1) and contained 1.4 µm tertiapin LQ (Estrada and Kaufman, 2018), 15 µm ML133 hydrochloride (Furst et al., 2014), 12 µm VU0134992 (Zhang et al., 2021), 13 µm VU590 (Lewis et al., 2009), and 2 µm repaglinide (Wang et al., 2018). There are components of K_ir_ expressed in rodent hippocampal PYR neurons that even the K_ir_-ct spares (e.g., K_ir_ 2.4, K_ir_ 3.3, and K_ir_ 4.2; Table 1), possibly accounting for the residual TGOT-sensitive current. To test this, we turned to use of Ba^2+^ (300 µm), known as a universal inhibitor of K_ir_ channels, along with other K^+^ channels. In this case, all subthreshold TGOT-S current was eliminated, as seen in a representative example (Fig. 3*C*) and in pooled data of current peak size (Fig. 3*D*). This finding demonstrates that the TGOT effect can be entirely occluded by preblocking the major components of basal membrane conductance, whether because of K_ir_ channels and/or other Ba^2+^-sensitive K^+^ channels. Non-K_ir_ K^+^ channels include the leak K^+^ channel (K_2P_; Lesage et al., 2000) and M channel (Yamada-Hanff and Bean, 2013), as considered below. The effects of the K_ir_ cocktail are less complete, yet still meaningful because of the K_ir_-specificity of the inhibitors. Taken together, these data imply that K_ir_ subtypes are indeed major targets of modulation.

### OXTR signaling reduces *I*_h_

**Figure 4.**
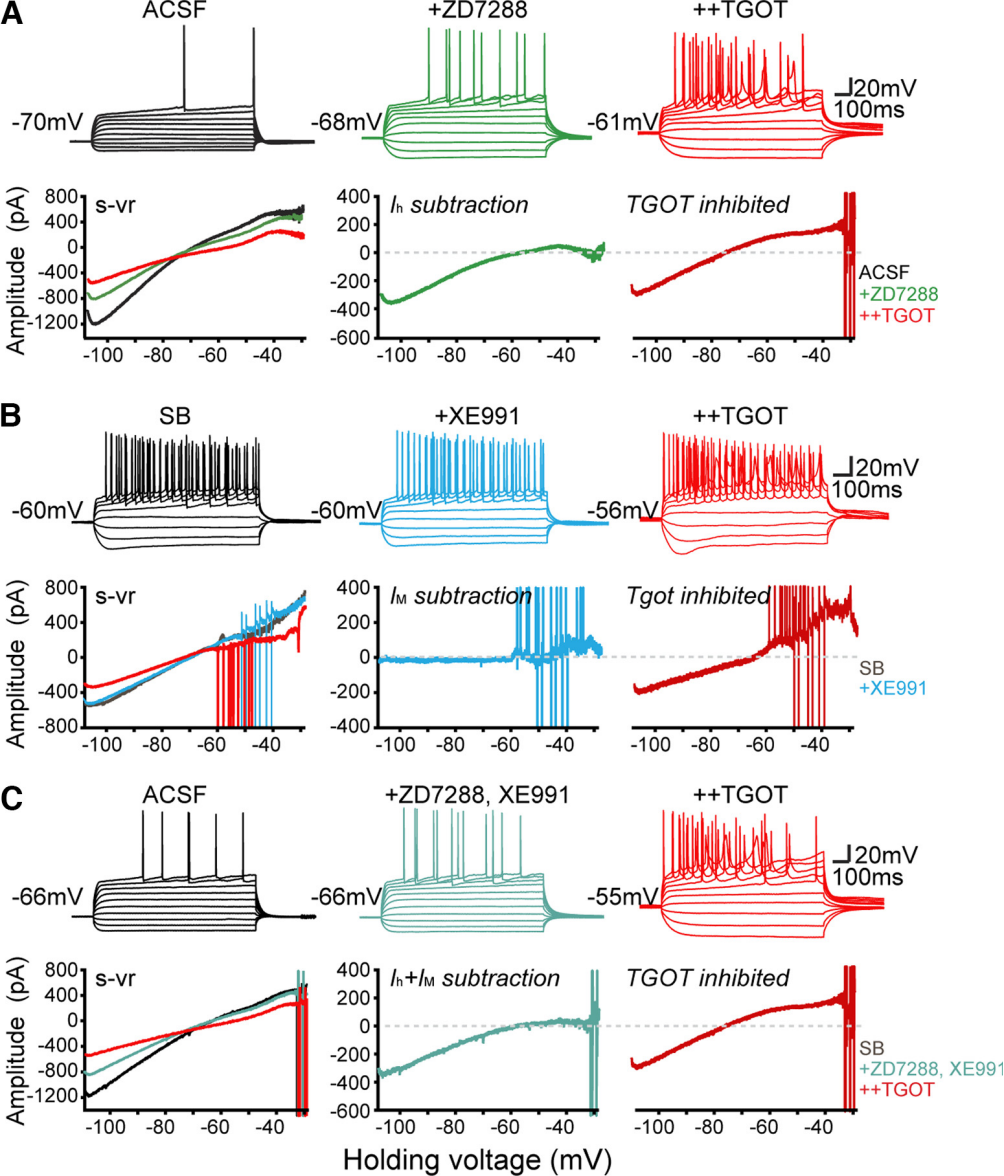
Blocking *I*_h_ or *I*_M_ or both neither mimic nor occlude TGOT's effects. ***A***, The effect of TGOT stimulation after blocking *I*_h_. Upper, Firing behavior of CA2 neuron in current-clamp step mode in control (black) and after serially adding 20 µm ZD7288 (green) and 600 nm TGOT (red). Lower, Effect of ZD7288, and additional TGOT on quasi-steady-state *I-V* relationship during s-vr in the same neuron. ZD7288-sensitive or TGOT-sensitive current was obtained by subtraction. ***B***, The effect of TGOT stimulation after blocking *I*_M_. Upper, Firing behavior of CA2 neuron in current-clamp step mode in control (black) and after serially added 10 µm XE991(blue) and TGOT (red). Lower, Effect of XE991, and additional TGOT on *I-V* relationship in the same neuron. XE991-sensitive or TGOT-sensitive current was obtained by subtraction. ***C***, The effect of TGOT stimulation after blocking both *I*_h_ and *I*_M_. Upper, Firing behavior of CA2 neuron in current-clamp step mode in control, ZD7288 and XE991, and additional TGOT. Lower, *I-V* relationships in the same neuron.

**Figure 5.**
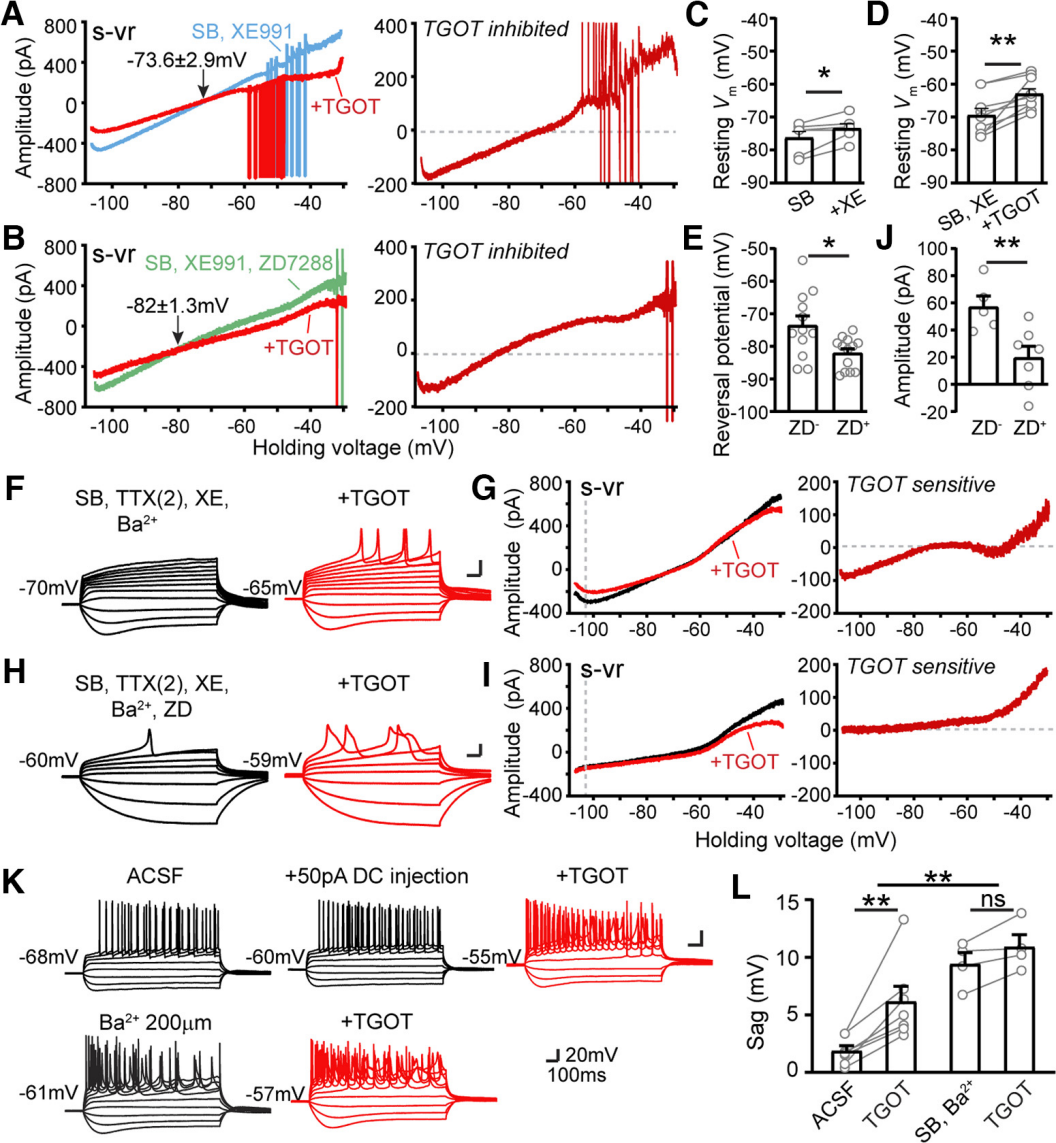
Involvement of *I*_h_ in TGOT inhibited conductance. ***A***, Effect of TGOT on steady-state *I-V* relationship with *I*_Kir_ and *I*_h_ intact. Currents recorded before (black) and during application of TGOT are plotted as a function of the command voltage. External solutions used contain: SB and 10 µm XE991. ***B***, Effect of TGOT on steady-state *I-V* relationship with *I*_Kir_ intact but *I*_h_ blocked. Currents recorded before (black) and during application of TGOT are plotted as a function of the command voltage. External solutions used contain: SB and XE991 and 20 µm ZD7288. TGOT-inhibited current was obtained by subtraction. ***C***, Pooled data of resting membrane potentials measured in SB + XE991. Gray dots represent individual cells. Bar graphs represent mean ± SEM: control −76.33 ± 1.98 and XE991 −73.5 ± 1.48 mV, *n* = 6, *p* = 0.0466. Paired two-tailed Student's *t* tests were used. ***D***, Pooled data of resting membrane potentials measured before and during TGOT application in external solution containing SB+XE991. Gray dots represent individual cells. Bar graphs represent mean ± SEM: control –69.5 ± 2.09 and TGOT −63 ± 1.62 mV, *n* = 9, *p* = 0.0038. Paired two-tailed Student's *t* tests were used. ***E***, Pooled data of reversal potential of TGOT-inhibited currents in external solution containing SB and 10 µm XE991, or containing SB, 10 µm XE991 plus 20 µm ZD7288. Gray dots represent individual cells. Bar graphs represent mean ± SEM: ZD^–^ −73.55 ± 2.89 and ZD^+^ −82.04 ± 1.33 mV, *n* = 12 and 13, respectively, *p* = 0.0116. Unpaired two-tailed Student's *t* tests were used. ***F***, Responses of CA2 neurons in current-clamp step mode before and during TGOT application in solution containing: SB, TTX(2), XE991, and 300 µm Ba^2+^. ***G***, Effect of TGOT on quasi-steady-state *I-V* relationship in the same neuron as ***F***. TGOT-sensitive current was obtained by subtraction. ***H***, Responses of CA2 neurons in current-clamp step mode before and during TGOT application in solution containing: SB, TTX(2), XE991, Ba^2+^, and ZD7288. ***I***, Effect of TGOT on quasi-steady-state *I-V* relationship in the same neuron as H. TGOT-sensitive current was obtained by subtraction. Demonstration that together, *I*_Kir_+*I*_h_ account for all the TGOT-sensitive current below −60 mV but not above −60 mV. ***J***, Pooled data of amplitudes of TGOT-inhibited currents measured at –105-mV command voltage in basal condition without or with ZD7288. Gray dots represent individual cells. Bar graphs represent mean ± SEM: ZD^–^ −57.04 ± 8.11 and ZD^+^ −24.41 ± 6.11 mV, *n* = 5 and 7, respectively, *p* = 0.0083. Unpaired two-tailed Student's *t* tests were used. ***K***, Block of K_ir_ channel mediated conductance accentuates *I*_h_-dependent sag. Upper, Firing behavior induced by current-clamp steps of a neuron before and during an additional DC injection, and with TGOT application. Lower, Firing behavior induced by current-clamp steps of a neuron in 200 µm Ba^2+^, and with TGOT application. ***L***, Pooled data of sag potential magnitude measured under various conditions. Gray dots represent individual cells. Bar graphs represent mean ± SEM: in ACSF 1.84 ± 0.44 mV and plus TGOT 6.14 ± 61.31 mV, *n* = 7, *p* = 0.0059; in SB and Ba^2+^ 9.42 ± 0.98 mV and plus TGOT 10.91 ± 1.05 mV. Paired two-tailed Student's *t* tests were used for within group analysis. Two-way ANOVA was used for the overall analysis, *F*_(1,14)_ = 15.42, *p* = 0.0015. ns: no significance, *p < 0.05, **p < 0.01, ***p < 0.001.

In the previous experiments, *I*_h_ and *I*_M_ were preblocked to allow focus on basally activated K^+^ channels, but we then specifically explored the possible involvement of these currents in OXTR signaling. We first examined the effect of OXTR activation after blocking either *I*_h_ or *I*_M_ or both (Fig. 4*A–C*). As previously mentioned, the *I*_h_ inhibitor ZD7288 suppressed an inward current that decreased to near zero with depolarization to –55 mV in CA2 OXTR^+^ neurons (Figs. 2*B*, 4*A*, lower green trace). In the continued presence of ZD7288, TGOT further depolarized *V*_m_, altered the shape of the AP and inhibited the inward current assessed at −105 mV (Fig. 4*A*), to an extent roughly like that seen without *I*_h_ blockade (Fig. 1*C*, lower right). Similarly, changes induced by TGOT application were preserved in the presence of the *I*_M_ inhibitor XE991 (Figs. 4*B*, 5*D*) or if *I*_h_ and *I*_M_ were simultaneously blocked (Fig. 4*C*). These results are consistent with K_ir_ channels being the primary target underlying OXTR-driven inhibition of resting membrane current (Fig. 3).

This conclusion was cross-checked against current clamp data. We found that XE991 mildly but significantly depolarized *V*_m_ relative to control (from −76.3 ± 2.0 to −73.5 ± 1.5 mV, *n* = 6, *p* = 0.0466 by paired *t* test; Fig. 5*C*), not different in magnitude from pooled data from a larger data set of unpaired samples (from −72.8 ± 0.91 mV, *n* = 26, to −69.7 ± 1.1 mV, *n* = 26, *p* = 0.2292 by unpaired *t* test) as shown in Figure 2*C*. This corroborates previous findings that only a limited proportion of M channels are open at rest but nonetheless participate in setting resting *V*_m_ of CA2 PYRs (Tirko et al., 2018; Robert et al., 2020).

Our experiments scrutinizing possible involvement of *I*_h_ in OXTR signaling yielded a more surprising result: TGOT reduced *I*_h_ according to multiple lines of evidence (Fig. 5). In the presence of ZD7288 to block *I*_h_, the reversal potential of TGOT-inhibited current (E_rev_) was displaced to more negative levels, from −73.6 ± 2.9 mV in control (*n* = 12; Fig. 5*A*) to −82.0 ± 1.3 mV in the additional presence of ZD7288 (*n* = 13; Fig. 5*B*). In pooled data (Fig. 5*E*), the displacement was significant (*p* = 0.0116 by unpaired *t* test). The ZD7288-driven 8–9 mV negative shift reflects the contribution of the nonselective cation channels that generate *I*_h_: by acting as a secondary target for OXTR-suppression, beyond K_ir_ channels, they keep E_rev_ positive to *E*_K_. This unexpected contribution is clearly revealed when *I*_h_ is pharmacologically blocked (Fig. 5*E*).

To determine the magnitude of the TGOT effect on *I*_h_, we eliminated K^+^ current using Ba^+^-containing external solutions and assessed the residual effect of TGOT without ZD7288 (Fig. 5*F*,*G*) or with ZD7288 present (Fig. 5*H*,*I*). Inclusion of ZD7288 reduced the TGOT-sensitive inward current from 57.0 ± 8.1 pA to −19.6 ± 8.5 pA (*n* = 5 and 7, respectively, *p* = 0.012; Fig. 5*G*, right, *I*, right, *J*). Reassuringly, the TGOT-sensitive current determined in the presence of Ba^2+^ displayed characteristics expected for *I*_h_, increasing with hyperpolarization negative to −60 mV (Fig. 5*G*, right) and disappearing altogether with further addition of ZD7288 (Fig. 5*I*, right).

While these results converge in indicating that TGOT partially suppresses *I*_h_, they appeared initially puzzling because of current clamp results (Figs. 1*B*, 5*K*): the hyperpolarization-induced sag potential induced by hyperpolarizing current pulses, small under basal conditions in CA2 PYRs (Chevaleyre and Siegelbaum, 2010; Srinivas et al., 2017; Tirko et al., 2018; Robert et al., 2020) grew larger after TGOT stimulation, not smaller as expected from diminution of *I*_h_. The sag remained small when the associated membrane depolarization was mimicked by injecting steady depolarizing current (DC; Fig. 5*K*). In contrast, preblocking *I*_Kir_ with Ba^2+^ greatly increased the sag potential and occluded the OXTR-induced enlargement of the sag (9.4 ±1.0 vs 10.9 ± 1.0 mV, *n* = 4, *p* = 0.0709 by paired *t* test; *F*_(1,14)_ = 15.42, *p* = 0.0015, two-way ANOVA; Fig. 5*L*). Our interpretation is that constitutive opening of K_ir_ channels shunts hyperpolarization of dendritic regions and thus hinders hyperpolarization-dependent *I*_h_ activation and sag. Upon exposure to Ba^2+^, hyperpolarization of *I*_h_-expressing membrane is more effective, sag is accordingly increased, and additional TGOT effects on sag are occluded (no *I*_Kir_ left to inhibit) or even counteracted (TGOT inhibition of *I*_h_).

The reduction of *I*_h_ resists TGOT-driven membrane depolarization, acting in partial opposition to simultaneous reduction of *I*_Kir_ and thus contributes to slowing the TGOT-mediated depolarization. On the other hand, *I*_h_ suppression synergizes with inhibition of K_ir_ in lowering the net membrane conductance (Fig. 9*D*). The functional outcome is thus enhancement of dendritic integration and promotion of excitability because of excitatory synaptic input (Fig. 9; also see Discussion). This fits with observation of a consistent, reversible TGOT-driven augmentation of spontaneous EPSCs (sEPSCs; Tirko et al., 2018; Fig. 10*C–E*), accompanying an increase in their amplitude (Fig. 10*C–E*). This is obvious in individual traces (Fig. 10*C*) and follows a time course (Fig. 10*D*) similar to that of elevated membrane Rin (Fig. 9*A*). A TGOT-induced increase of the amplitude of postsynaptic response was seen in every recording of sEPSCs over the range of TGOT concentrations from 10 up to 600 nm (Fig. 10*C*, upper panel, *E*, *n* = 13, open symbols) and of miniature EPSCs recorded with TTX present (Fig. 10*C*, lower panel, *E*, *n* = 2, filled symbols); net *p* = 0.0003 by paired *t* test. We suggest that responses to neurotransmitter quanta are augmented by altered intrinsic properties and elevated synaptic integration (Fig. 9*D*, bottom middle icons).

### *I*_K2P_, *I*_NALCN_, and Cl^–^ channels are likely spared by OXTR signaling

**Figure 6.**
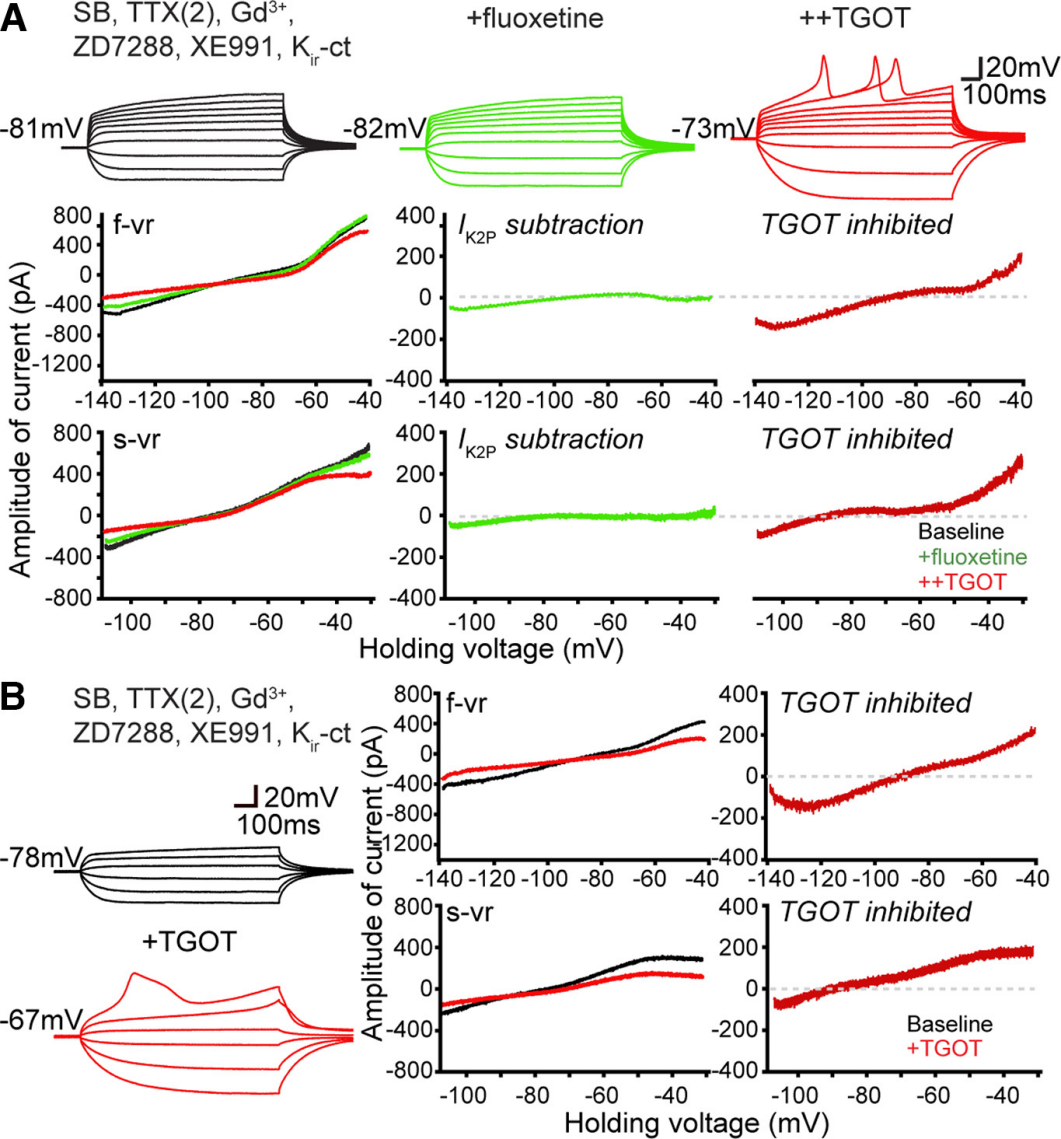
Testing for involvement of *I*_K2P_ and Cl^–^ channels in TGOT inhibited conductance. ***A***, Responses of a CA2 OXTR neuron recorded in a baseline solution (black) and after serially adding 100 µm fluoxetine to define contribution of *I*_K2P_ (light green), and TGOT-sensitive current (red). Upper, In current-clamp step mode. Lower, Steady-state currents obtained with f-vr and s-vr of the same neuron. Current component sensitive to fluoxetine or TGOT was obtained by subtraction. ***B***, Exclusion of chloride current as significant contributor to TGOT-sensitive current. Responses of a CA2 neuron recorded before (black) and during TGOT application (red), using a high Cl^–^ internal solution (in mm; 40 CsCl, 90 K-gluconate, 1.8 NaCl, 1.7 MgCl_2_, 3.5 KCl, 0.05 EGTA, 10 HEPES, 2 Mg-ATP, 0.4 Na_2_-GTP, and 10 phosphocreatine, pH to 7.2 with CsOH) containing QX 314. Left, In current-clamp step mode. Right, Steady-state currents obtained with f-vr and s-vr of the same neuron. TGOT-sensitive current was obtained by subtraction.

Next, we asked whether certain ion conducting pathways might be ruled out as contributors to OXT-driven changes in intrinsic properties. We considered the leak potassium channels known as K_2P_, which are spared by the K_ir_-ct but responsive to Ba^2+^ block. Exemplified by TREK-1, which is upregulated in schizophrenia model mice (Piskorowski et al., 2016) and exerts behavioral effects on social memory (Donegan et al., 2020), this class of channels is susceptible to block by fluoxetine (Prozac; Kennard et al., 2005). As an initial test of K_2P_ channel involvement, we exposed CA2 PYRs to 100 µm fluoxetine in the presence of ZD7288 and K_ir_-ct (Fig. 6*A*). The fluoxetine caused only a very small incremental conductance change, consistent with evidence that wild-type mouse CA2 PYRs display little basal K_2P_ conductance (Piskorowski et al., 2016). Additional TGOT application still depolarized CA2 neurons and closed a conductance reversing at *E*_K_, indicating that a fluoxetine-sensitive component of *I*_K2P_ was not essential for TGOT modulation and likely spared.

A major caveat to our approach to isolation of current components is its dependence on pharmacology. This issue came up earlier in comparing effects of the K_ir_-blocking cocktail, which partially spares some K_ir_ channels, with blockade by Ba^2+^, which eliminates all K_ir_ current but also has potential for affecting K_2P_ channels (TWIK, TREK, TASK, THIK, and TALK channel families). Data from RNA-seq indicates that the K_2P_ channel subtypes detectably expressed in the dHP are TWIK-1 (K_2P_1.1; Goldstein et al., 2001), which is expressed throughout the entire HP but encodes an inactive channel that fails to generate current, and TREK-1 (K_2P_2.1), which is expressed at higher level in CA2 PYRs than in other hippocampal regions (Talley et al., 2001). Accordingly, we believe that blocking TREK-1 with fluoxetine should remove the majority, if not all, *I*_k2P_ in the CA2 PYRs we studied. However, roles for other K_2P_ channels cannot be fully excluded.

Some of our recordings were performed in the presence of gadolinium (Gd^3+^), a potent inhibitor of both voltage-gated Ca^2+^ channels, including T-type Ca^2+^ channels (Biagi and Enyeart, 1990) as well as sodium leak channels (*NALCN*; Lu et al., 2007; Ren, 2011) that serve as powerful effectors of peptide regulatory signaling (Lu et al., 2009; Ren, 2011). Nonetheless, the TGOT-sensitive current was not noticeably different with inclusion of Gd^3+^ (Fig. 6*A*).

Chloride channels are sometimes overlooked in the press of investigating multiple cation channels, though their functional role in regulating neuronal excitability and AP waveform is substantial (Lin et al., 2012). As a generic test of possible involvement of Cl^–^ channels in TGOT inhibited membrane conductance, we elevated intracellular Cl^–^ in the internal solution to move *E*_Cl_ away from *E*_K_ (Fig. 6, legend), allowing us to disambiguate any contribution of Cl^–^ conductance. If TGOT had induced a lowering of basal Cl^–^ conductance, the *I-V* curve of TGOT-inhibited current would have shifted positively toward *E*_Cl_, away from *E*_K_. Instead, we found that *E*_rev_ of TGOT-inhibited current remained close to *E*_K_ even with high internal Cl^–^, ruling out resting chloride channels as a significant target of TGOT modulation.

Altogether, these results reinforced the conclusion that TGOT-inhibited conductance in CA2 OXTR^+^ PYRs was mainly mediated by K_ir_ channels. TGOT also reduces *I*_h_, but appears to spare *I*_K2P_, *I*_NALCN_ and *I*_Cl_.

### OXTR signaling activates TTX-R sodium current for pacemaking

**Figure 7.**
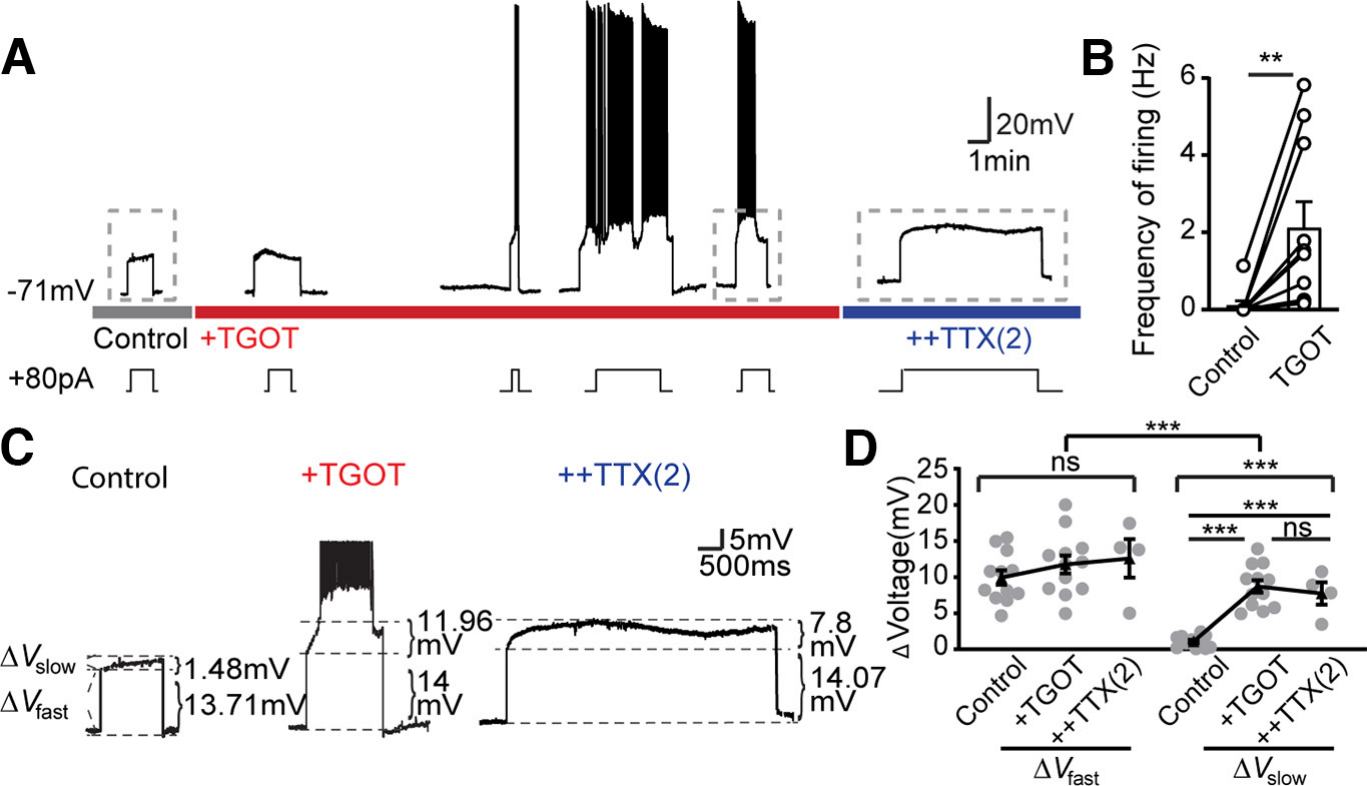
TGOT activates TTX-R sodium current for pacemaking. ***A***, Current clamp recording of a CA2 OXTR neuron under different conditions in a basal solution while serially adding TGOT and TTX (2 µm). To facilitate AP firing, we injected positive current pulses of amplitude needed to depolarize beyond −60 mV but below −55 mV under basal conditions for each neuron, then maintained this input for the rest of the recording. ***B***, Pooled data of AP frequencies induced by step current injection before and during Tgot application. Data are presented as mean ± SEM: control 0.11 ± 0.11 Hz, TGOT 2.12 ± 0.67 Hz, *n* = 10, *p* = 0.0096. Paired two-tailed Student's *t* tests were used. ***C***, Zoomed-in view of traces denoted by dashed boxes in ***A***. Current injection induced a near-instantaneous *V*m change, determined by cell membrane resistance, defined as Δ*V*_fast_, and a slowly developed depolarization, termed Δ*V*_slow_, likely because of opening of voltage gated cation channels. ***D***, Pooled data of both Δ*V*_fast_ and Δ*V*_slow_ in control, TGOT, and TTX(2). Gray dots represent individual cells. Black dots and error bars denote mean ± SEM: Δ*V*_fast_ control 9.93 ± 0.99 mV, TGOT 11.75 ± 1.24 mV, and TTX(2) 12.573 ± 2.66 mV; Δ*V*_slow_ control 1.08 ± 0.23 mV, TGOT 8.69 ± 0.85 mV, and TTX(2) 7.72 ± 1.55 mV. Two-way ANOVA was used for between group analysis, *F*_(1,43)_ = 43.72, *p* < 0.0001. One-way ANOVA was used for within group analysis: Δ*V*_fast_ *F*_(2,25)_ = 0.816, *p* = 0.4535. Δ*V*_slow_ *F*_(2,25)_ = 32.43, *p* < 0.001; *post hoc* Tukey's tests used for multiple comparisons control versus TGOT *p* < 0.0001, control versus TTX(2) *p* = 0.0002, TGOT versus TTX (2) *p* = 0.7098. ns: no significance, *p < 0.05, **p < 0.01, ***p < 0.001.

We turned next to the underlying basis of the spontaneous oscillatory variation in *V*_m_, evident in multiple figures. Such activity derives from dynamic current changes over the “pacemaker range” of membrane voltage, spanning from −65 mV (the lowest trough seen during spontaneous firing) to −49 mV (∼5 mV negative to the spike threshold during activity). In CA1 PYR subjected to mAChR modulation, Yamada-Hanff and Bean showed that the dominant inward excitatory current and the main driver of repetitive firing is the persistent, TTX-S sodium current, *I*_NaP, TTX-S_ (Yamada-Hanff and Bean, 2013). To explore the possible contribution of *I*_NaP, TTX-S_ in CA2 PYRs (Fig. 7), we examined the subthreshold *V*_m_ change during repeated pulses of DC of fixed amplitude (+80 pA in this exemplar), adjusted to bring the initial *V*_m_ to slightly above −60 mV. The evoked depolarization skipped over the events that support the initial depolarization from rest as described earlier. Driven to the pacemaker voltage range, CA2 neurons rarely showed spiking activity basally (Fig. 7*A*,*B*) but responded to TGOT with an additional slow depolarization and oscillatory burst-like firing (from 0.11 ± 0.11 to 2.12 ± 0.67 Hz, *n* = 10, *p* = 0.0096). Exposure to TTX(2) (marked with blue bar) abolished the spikes as anticipated, but unexpectedly spared the slow depolarization as seen in both the exemplar recording (Fig. 7*C*) and pooled data (Fig. 7*D*). We analyzed this by measuring a rapid *V*_m_ change, Δ*V*_fast_, generated by the current step, and a slower change, Δ*V*_slow_, because of the dynamic increase of voltage-gated inward current. Whereas successive exposure to TGOT and TTX(2) had no significant impact on the Δ*V*_fast_ (Fig. 7*C*,*D*), Δ*V*_slow_ was strongly elevated by TGOT, an increase not prevented by subsequent application of TTX(2) (Fig. 7*C*,*D*). This TTX-R slow depolarization echoed spikelet-like activity evoked by TGOT stimulation in earlier experiments wherein *I*_NaP, TTX-S_ had been eliminated in TTX(2) (Figs. 5*C*, 6*A*). In turn, the TTX-R depolarization in current-clamp was analogous to the TGOT-induced inward current in Figure 5*H*,*I*. We conclude that CA2 PYRs use regenerative currents over the pacemaker range but rely on an inward current component other than *I*_NaP, TTX-S_ and thus differ from CA1 PYRs (Yamada-Hanff and Bean, 2013).

**Figure 8.**
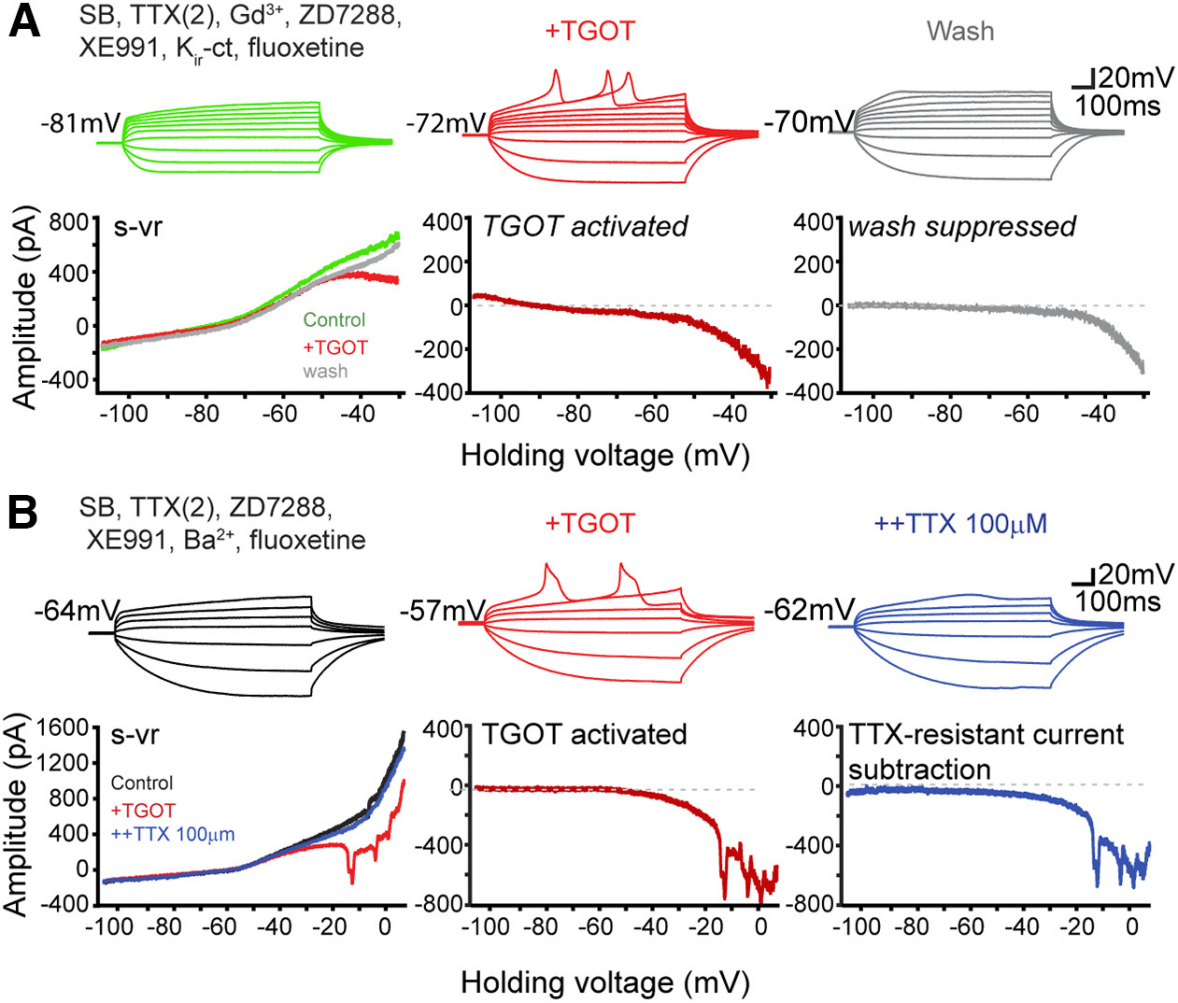
TGOT activates an inward current in CA2 neurons that requires high [TTX] for blockade. ***A***, Responses of a CA2 neuron in current-clamp step mode in a baseline solution (black) while serially adding TGOT (red) and 100 µm TTX (purple). ***B***, Steady-state currents obtained with f-vr and s-vr (from −108 to +8 mV at 20 mV/s) of the same neuron under each condition. TGOT-enhanced and 100 µm TTX-inhibited currents were obtained by subtraction.

Interestingly, next-generation RNA sequencing (RNA-seq) reveals restricted expression of mRNA encoding a TTX-R voltage-gated channel Na_V_1.9 in the CA2 pyramidal neurons that is barely detectable in CA1 or CA3 PYRs (Cembrowski et al., 2016). Encoded by *scn11a*, Na _V_1.9 displays an IC_50_ for TTX of 40 µm (Rush and Waxman, 2004). To look for such TTX-R sodium current (*I*_Na, TTX-R_), we recorded CA2 neurons under conditions where pharmacological blockers inhibited contributions of synaptic inputs, *I*_NaP, TTX-S_, NALCN channels, *I*_h_, *I*_M_, *I*_Kir_, and *I*_K2P_ pathways (Fig. 8). TGOT-induced current changes at strongly negative resting potentials were eliminated as expected (Fig. 8*A*, lower s-vr traces), but over the pacemaker range, a TGOT-activated inward current (Fig. 8*A*, lower TGOT-activated red trace) and corresponding repetitive spontaneous depolarization activity (Fig. 8*A*, upper TGOT red traces) were nonetheless induced. Upon removal of TGOT (wash, gray traces), the spontaneous depolarizations and TTX-R inward current largely disappeared (Fig. 8*A*, gray traces).

**Figure 9.**
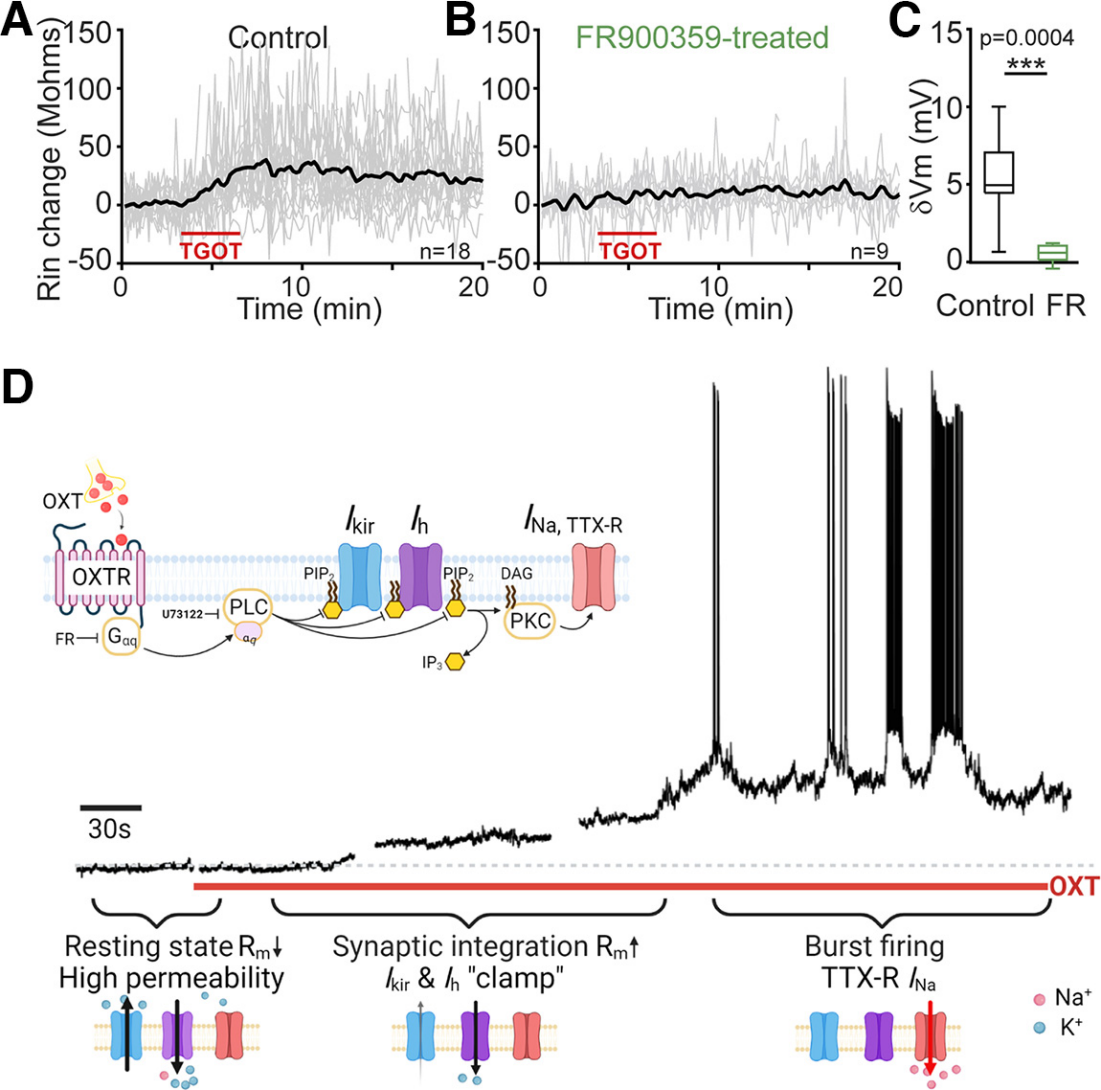
Functional logic of OXTR regulation of CA2 PYR excitability. ***A–C***, Evidence for G_αq_ involvement based on use of FR900359 (FR), inhibitor of G_αq_ signaling. ***A***, Membrane resistance (Rin) change during TGOT application in control condition; *n* = 18. ***B***, Rin change induced by TGOT application on pretreatment with FR; *n* = 9. ***C***, Pooled data showing that inhibition of G_q_ signaling also blocks the depolarizing response to OXTR activation by TGOT; *p* = 0.0004 by paired *t* test. ***D***, Schematic depiction of ensemble of ion channel mechanisms downstream of OXTR, G_αq_ signaling and PLC, whose involvement is supported by U73122 data (Tirko et al., 2018). This signal transduction concatenates distinct components of the response, including inhibition of K_ir_ (*I*_kir_) and HCN (*I*_h_) channels, along with enhancement of TTX-R voltage-gated sodium current (*I*_Na, TTX-_***_R_***). These components, all voltage-dependent, dominate over different voltage ranges, so their mobilization impacts electrogenesis at early and later stages of the response as shown below. Depolarization in early phase activates *I*_Na, TTX-R_ in later phase of continual bursting; *p < 0.05, **p < 0.01, ***p < 0.001.

Next, to better characterize the TGOT-activated current, we extended the s-vr to +8 mV while maintaining the slow ramp speed (Fig. 8*B*). Even in the presence of TTX(2), with Ba^2+^ included as a generic K^+^ channel blocker, TGOT activated a large, voltage-dependent inward current that triggered repetitive activity in current clamp (also registered as poorly controlled downward spikes in s-vr recordings) and a voltage-dependent inward current, steeply rising over the pacemaker range. Subsequent elevation of [TTX] to 100 µm significantly eliminated both the repetitive activity and inward current, consistent with participation of a TTX-R sodium current over the pacemaker range. TTX-R sodium channels are known to contribute to peptide responses and bursting pacemaker potentials in molluscan neurons (Barker and Gainer, 1975a, b; Barker et al., 1975; Barker and Smith, 1976; Nambu and Scheller, 1986; van Soest and Kits, 1998) and peripheral mammalian neurons (Raggenbass et al., 1991; Raggenbass and Dreifuss, 1992; Alberi et al., 1997; Rush and Waxman, 2004; Baker, 2005; Ostman et al., 2008; Jiang et al., 2014; Baker and Nassar, 2020), but finding a role in peptide modulation of hippocampal neurons is novel (Fig. 9, See Discussion).

**Figure 10.**
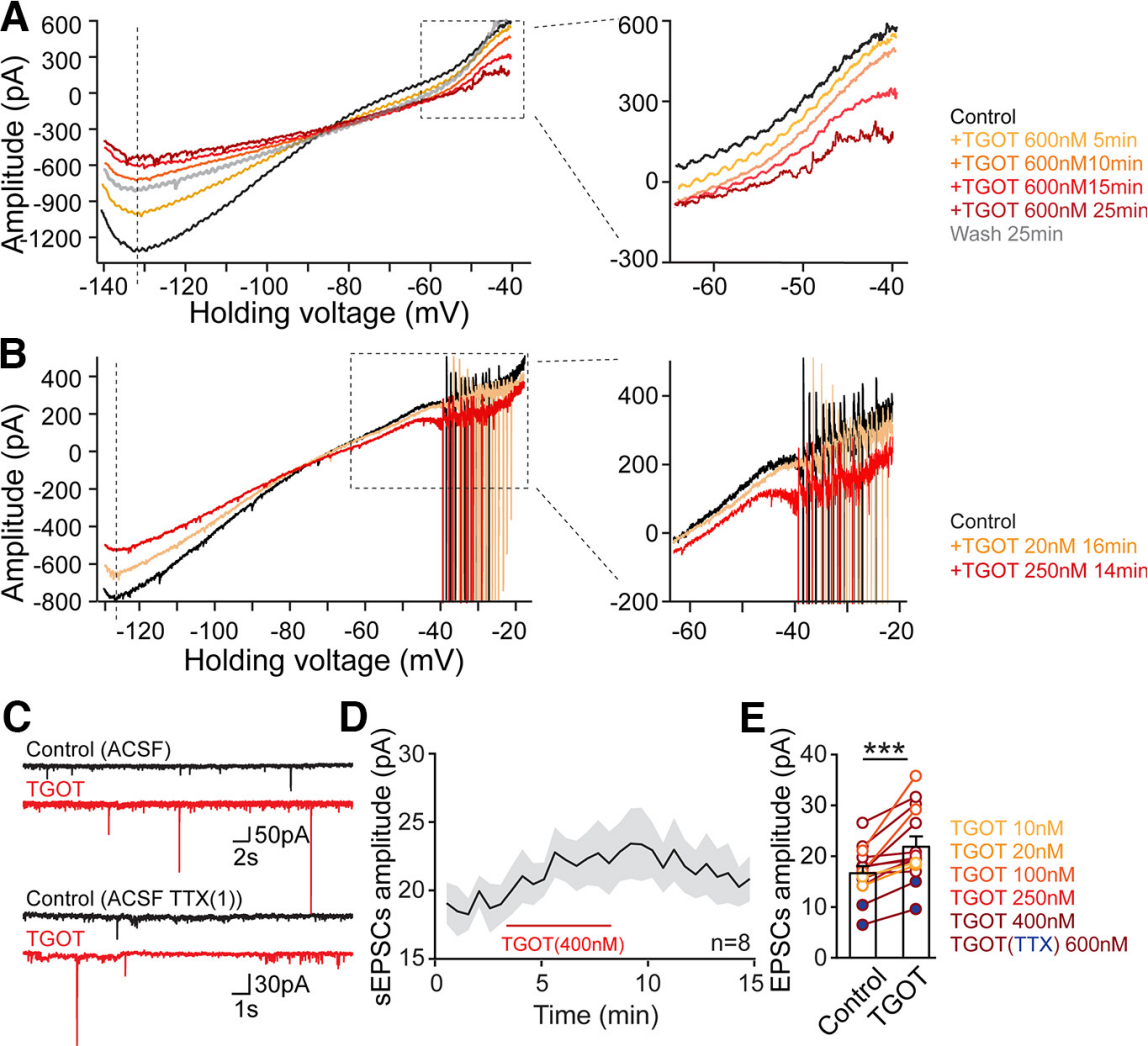
*I*_Kir_ and *I*_h_ are more sensitive to TGOT stimulation relative to *I*_Na, TTX-R_. ***A***, Ionic mechanisms underlying OXT modulation in CA2 PYRs compared on a temporal basis, *n* = 5. ***B***, Ionic mechanisms under OXT modulation in CA2 PYRs compared on a basis of concentration, *n* = 4. ***C***, Sample traces recorded at −70 mV showing spontaneous EPSCs (upper panel) and miniature EPSCs (lower panel) before (control, black trace) and after application of TGOT (TGOT, red trace). ***D***, Time course of increase in sEPSC amplitude during 400 nm TGOT application, *n* = 8. ***E***, Pooled data showing TGOT-induced increase in EPSC amplitudes, recorded in the absence of TTX (open circles, *n* = 13, sEPSC frequency data previously published; Tirko et al., 2018), and in the presence of TTX (filled circles, *n* = 2). Overall, *p* = 0.0003 by paired *t* test; *p < 0.05, **p < 0.01, ***p < 0.001.

Finally, we explored whether the currents we identified have differing sensitivity to the duration of exposure or concentration of OXTR agonist. Holding the TGOT concentration (600 nm) fixed, we found that the activation of *I*_Na, TTX-R_ takes longer to develop than the inhibition of *I*_h_ and *I*_kir_ (Fig. 10*A*). Likewise, when we varied [TGOT], testing concentrations of 10, 20, 100, or 250 nm (Fig. 10*B*), *I*_h_ and *I*_kir_ were responsive to lower concentrations of TGOT (10 or 20 nm), whereas the change in I_Na, TTX-R_ only appeared at higher concentrations and could be reversed following removal of the agonist. The mechanism underlying this difference requires further study: given the dependence of burst firing on PKC activation downstream of OXTRs (Fig. 9, Tirko et al., 2018), one possibility worth considering is a PKC-driven recruitment of covert channels (Strong et al., 1987; Conn et al., 1989; White and Kaczmarek, 1997; Groten and Magoski, 2015).

## Discussion

The ionic mechanisms underlying OXT control of dCA2 PYR excitability are of particular interest because these neurons play essential roles in generation of brain oscillations and social memory (Hitti and Siegelbaum, 2014; Raam et al., 2017; Oliva et al., 2020); conditional knock-out of OXTRs in this region impairs social recognition (DeVries et al., 1997; Ferguson et al., 2001; Wang et al., 2018). Our experiments show that activation of OXTR (1) drives depolarization of CA2 PYRs by closing K_ir_ channels that contribute outward current at rest; (2) reduces the *I*_h_ conductance, providing a hyperpolarizing counterforce to help stabilize the membrane potential that works alongside *I*_Kir_ inhibition to increase membrane resistance and favor dendritic integration; (3) enables the voltage-dependent recruitment of a TTX-R Na^+^ current that helps further depolarization and promotes rhythmic firing. This novel array of OXTR-stimulated ionic mechanisms operates in close coordination, strongly controls excitability and underpins OXT-induced burst firing, a key factor in CA2 PYRs' contribution to hippocampal information processing and broader influence on brain circuitry (Dudek et al., 2016; Jurek and Neumann, 2018; Grinevich and Neumann, 2021). We next consider the individual ion channel targets, discuss their functional relation to synaptic inputs and outputs and how our findings fit within a broader pattern of peptide neuromodulation.

### Sculpting CA2 activity with OXTR targets from an evolutionarily conserved palette

OXT-/vasopressin-like nonapeptides exemplify peptide neuromodulators conserved across invertebrate and vertebrate taxa (Bargmann and Marder, 2013; Jurek and Neumann, 2018; Theofanopoulou et al., 2021). OXT generally depolarizes target cells by inducing a net inward current, with varied ionic mechanisms suggested (Owen et al., 2013; Jiang et al., 2014; Tang et al., 2014; Briffaud et al., 2015; Tirko et al., 2018; Maniezzi et al., 2019; Hu et al., 2020, 2021; Zhang et al., 2021). CA2 PYRs provided a suitable testbed for evaluation of potential target mechanisms with interleaved current clamp and voltage clamp recordings and well-characterized pharmacological agents. By first blocking synaptic inputs, and then ionic pathways, one or more at a time, we were able to occlude the impact of OXTR stimulation by preblockade of individual pathways. Our analysis of the slowly changing currents that control the leadup to burst firing complements studies of much larger currents flowing during the burst firing itself (Robert et al., 2020).

### Importance of k_ir_ modulation in the initial depolarization

OXTR activation significantly increases the Rin of CA2 PYRs, suggesting a closing of ion channels such as K^+^ channels open at rest (Tirko et al., 2018). An early candidate, shutting off M current channels, can now be ruled out as the dominant mechanism on several grounds. First, CA2 neurons' initial depolarization starts below −70 mV, yet *I*_M_ is undetectable below −60 mV (Fig. 2*B*). Second, TGOT-inhibited currents show inward rectification, inconsistent with *I*_M_ but in line with inhibition of K_ir_ channels. Third, closing *I*_M_ with XE991 failed to occlude further depolarization by TGOT. These data rule out *I*_M_ inhibition as dominant in OXTR-mediated depolarization but leave room for modulation of burst firing by the *I*_M_-activator retigabine (Tirko et al., 2018).

We find that the OXTR-induced depolarization is largely driven by the closing of inwardly rectifying K_ir_ channels. Involvement of *I*_Kir_ accounts for the voltage dependence of the TGOT-sensitive current and its blockade by Ba^2+^ and by a cocktail of organic K_ir_ antagonists, a reassuring alignment of pharmacological approaches. Though OXT was not known to close K_ir_ channels in the HP, this was implicated in neurons of amygdala (Hu et al., 2020) and spinal cord (Jiang et al., 2014). The participation of PIP_2_ depletion is suggested by effects of the G_q/11_ blocker FR900359 and PLC-β1 inhibition with U73122 (Tirko et al., 2018; Fig. 9).

### Functional implications of *I*_h_ downregulation in conjunction with k_ir_ inhibition

Multiple lines of evidence indicated that OXTR activation also reduced *I*_h_. The reversal potential of TGOT-inhibited current (E_rev_) was shifted by −8 mV to more negative levels by preblocking *I*_h_ with ZD7288, from ∼−74 mV in control to ∼82 mV, reflecting *I*_h_ acting as an additional target for OXTR-suppression beyond K_ir_ channels. Likewise, in the absence of *I*_Kir_, the TGOT-sensitive current at a negative test potential was ∼40 pA smaller with ZD7288 present than without. These results converge in indicating that TGOT partially suppresses *I*_h_. Suppression of *I*_h_ has been previously reported as a mechanism for sculpting short-term synaptic plasticity (Heys et al., 2012; Sparks and Chapman, 2014).

What is unprecedented and apparently paradoxical is to find reduction of both *I*_Kir_ and *I*_h_, operating in parallel. Individually, these modulatory effects would depolarize or hyperpolarize a target neuron, potentially cancelling other out or at least generating variability of TGOT effects. Indeed, we occasionally observed an early hyperpolarization that precedes depolarization (Eyring, 2020), although the more common finding was an increase in Rin R_m_ even before an appreciable change in V_m_ (Fig. 9*A*). Our interpretation is that simultaneous reduction of both *I*_Kir_ and *I*_h_ would synergize in altering the low basal R_m_ and a reluctance to respond to synaptic inputs, features that distinguish CA2 PYR from PYRs in neighboring CA3 and CA1 (Chevaleyre and Siegelbaum, 2010; Robert et al., 2020). This functional rationale was anticipated by computer modeling of PYR dendrites and the interplay between *I*_Kir_ and *I*_h_ conductances (Day et al., 2005). Thus, OXTR activation would render CA2 PYRs more responsive to synaptic inputs, joining with altered intrinsic properties to promote spiking activity (Fig. 9*D*). We found evidence that spontaneous excitatory synaptic events grew consistently larger on OXTR activation, even when presynaptic spiking was blocked with TTX, consistent with enhanced dendritic integration of synaptic input (Fig. 10*C–E*).

### TTX-R sodium channels as drivers of OXTR-driven spontaneous bursting

After OXTR-dependent CA2 PYR depolarization is initiated, a further step is the voltage-dependent engagement of TTX-R Na^+^ channels. These channels were reflected by repetitive pacemaker activity, oscillatory potentials, even after blockade of *I*_M_, *I*_h_, *I*_Kir_, *I*_K2P_, and *I*_Ca_. Although not easy to study because of imperfect voltage control, the TTX-R Na^+^ channels provided a voltage-dependent inward current (*I*_Na, TTX-R_) reliably potentiated by TGOT, not blocked by 1–2 µm TTX (but sensitive to TTX at high concentration (100 µm). *I*_Na, TTX-R_ was critical for the progressive shifting of the membrane potential and the eventual induction of burst firing.

The closest precedent for OXTR-evoked *I*_Na, TTX-R_ is the TTX-R voltage-gated Na^+^ currents in spinal cord neurons (Jiang et al., 2014), brainstem vagal neurons (Raggenbass and Dreifuss, 1992) and possibly the TTX-R *I*_Na_ evoked by BDNF in HP CA1 neurons (Kafitz et al., 1999; Blum et al., 2002). Peptide-evoked TTX-R Na^+^ currents have been implicated in reproductive and/or social behavior across various phyla. In molluscan neurons, Egg Laying Hormone activates an “*I*_IN_” supported by Na^+^ entry and resistant to 60 µm TTX (van Soest and Kits, 1998); vasopressin and OXT activate a voltage-dependent Na^+^ current underlying bursting pacemaker potentials (Barker and Gainer, 1975a, b; Barker et al., 1975; Barker and Smith, 1976); and conopressin, an OXT/vasopressin homolog, activated pacemaker-generating voltage-gated Na^+^ currents (van Soest and Kits, 1998). In mammals, TTX-R voltage-dependent inward currents are activated in brainstem neurons by peptides ranging from arginine-vasopressin (AVP; Raggenbass et al., 1991) to OXT (Raggenbass and Dreifuss, 1992). In spinal cord nociceptive sensory neurons, the *I*_Na, TTX-R_ helps set thresholds for excitability by modulating both the resting potential and responses to subthreshold stimuli; *I*_Na, TTX-R_ undergoes modulation via GPCR-mediated signaling (Rush and Waxman, 2004; Baker, 2005; Ostman et al., 2008; Baker and Nassar, 2020) and cannabidiol (Zhang and Bean, 2021). Our results reinforce these earlier findings and suggest that activation of TTX-R Na^+^ channels may be a widespread effector of neuromodulatory signaling across evolution. Encouraging future work on the molecular basis of *I*_Na, TTX-R_, we find both Na_V_1.8 and Na_V_1.9 mRNA in CA2 neurons using RNAscope, largely in line with data from HippoSeq (Cembrowski et al., 2016), thus providing candidate TTX-R sodium channels.

### Circuit implications of OXTR-stimulated changes in CA2 PYR intrinsic properties

Our data show that CA2 pyramidal neurons sharply differ from their counterparts in area CA1 in how dynamic changes in membrane potential are sculpted. In CA1 PYR, an *I*_NaP, TTX-S_ provides a dynamic current at subthreshold potentials and thus plays a dominant role in pacemaking (Yamada-Hanff and Bean, 2013). We exclude OXTR-modulation for pathways such as *I*_K2P_, *I*_NALCN_, and *I*_Cl_, whose involvement was directly probed. Yet other channels such as TRPV1 channels (Zhang et al., 2021), L-type Ca^2+^ channels (Maniezzi et al., 2019), nonselective cation channel and the Na^+^-Ca^2+^ exchanger (Tang et al., 2014) and TRPC-like channels (Briffaud et al., 2015) were not obvious in our experiments. The dominant ion mechanisms and their varied dependence on time and concentration of exposure create two phases to the OXTR response, separated according to voltage range. First, acute responsiveness to synaptic input and facilitated synaptic integration. Second, full-blown burst firing driven by powerful intrinsic mechanisms, but further shaped by inhibitory feedback (Tirko et al., 2018). In turn, the grouping of spikes in bursts influences short-term synaptic plasticity at output synapses, and thus the impact of CA2 PYRs on downstream target networks.
